# 326. Meeting an unmet need - impact of a Complex Fungal Infection Clinic in the UK

**DOI:** 10.1093/ofid/ofad500.397

**Published:** 2023-11-27

**Authors:** Catherine Dominic, Hamish Houston, Abhishek Das, Neil RH Stone

**Affiliations:** Barts and the London School of Medicine, London, England, United Kingdom; Hospital for Tropical Diseases, London, England, United Kingdom; Hospital for Tropical Diseases, London, England, United Kingdom; University College London Hospitals , London, England, United Kingdom

## Abstract

**Background:**

The WHO recently set out priority fungal pathogens of global importance which were previously neglected in terms of awareness & funding^(1)^. Accurate diagnosis of fungal infection is a challenge & mortality remains high^(2)^ as expertise and dedicated mycology services are lacking globally. Our centre provides one of the UK’s first complex fungal infection clinics, established in 2019. We sought to describe the patients attending this clinic, as a step towards understanding the landscape of clinical mycology in the UK.

**Methods:**

Electronic health record systems were searched for all patients attending our clinic over 4 years from 01/04/19-01/04/23. Patients referred for consideration of a fungal diagnosis were included in the analysis. Invasive fungal infections (IFIs) were classified as per EORTC/MSGERC guidance.^(3)^

**Results:**

80/407 patients attending our clinic were referred for consideration of a fungal diagnosis. 31 (39%) were male. Median age was 53 (IQR 38-62). Most referrals (59/80; 74%) were received from within our institution. 41 (51%) had a fungal diagnosis confirmed prior to review. 28 were treated for invasive fungal infections (IFIs), 9 for chronic mycoses (including 5 chronic pulmonary aspergillosis & 2 mycetoma), 28 for superficial mycoses (20 recurrent mucocutaneous infections & 8 onychomycoses) & 15 had no evidence of fungal infection. Of the IFI: 16 were proven; 8 probable; 2 possible IFI & 2 did not meet diagnostic criteria. 7 IFI were caused by *Aspergillus*, 8 by *Cryptococcus* & 2 each by *Coccidiodes, Candida* & *Histoplasma*. The IFIs mainly affected the upper & lower respiratory tract (17/28). 20/80 (25%) were immunosuppressed (commonest causes: diabetes; HIV; steroids; malignancy & chemotherapy). On average (median), patients visited clinic 4 times (IQR 2-7) & received 2 different antifungal drugs (IQR 1-3). The most common side-effects prompting a change in therapy were visual changes, rash & diarrhoea.

**Conclusion:**

Our data illustrate the need for dedicated follow-up for patients with complex fungal infections. With several new antifungal drugs currently undergoing clinical trials, complexity in therapeutic decision-making will only increase.^(4)^ Establishing centralised complex fungal infection clinics helps to build diagnostic & clinical capacity in mycology.

**Disclosures:**

**Hamish Houston, BA MBBCh**, Gilead Sciences: Grant/Research Support **Neil RH Stone, MD PhD**, Gilead: Advisor/Consultant|Gilead: Grant/Research Support|Gilead: Honoraria|Pfizer: Honoraria|Shionogi: Honoraria

